# Factors associated with body mass index in a cohort of older adults: Structural equation modeling analysis

**DOI:** 10.1371/journal.pone.0305878

**Published:** 2024-07-18

**Authors:** Bruno de Freitas Camilo, Nayara Gomes Nunes Oliveira, Alisson Fernandes Bolina, Lucas Lima Galvão, Sheilla Tribess, Jair Sindra Virtuoso Júnior

**Affiliations:** 1 Department of Body and Human Movement, State University of Minas Gerais, Passos, Minas Gerais, Brazil; 2 Postgraduate Program in Health Care, Federal University of Triângulo Mineiro, Uberaba, Minas Gerais, Brazil; 3 Postgraduate Program in Nursing, University of Brasília, Brasília, Distrito Federal, Brazil; 4 Physical Education and Sports Center, Postgraduate Program in Physical Education, Federal University of Espírito Santos, Vitória, Espírito Santo, Brazil; 5 Department of Sports Sciences Postgraduate, Program in Physical Education, Federal University of Triângulo Mineiro, Uberaba, Minas Gerais, Brazil; University of Jyvaskyla, FINLAND

## Abstract

**Background:**

Considering the interrelationship between the multiple determinants of nutritional status, analyses are needed to estimate direct and mediated effects between the variables that influence body mass index (BMI) in older adults. We aimed to verify the direct and indirect effects of sociodemographic, behavioral and health conditions on the BMI of older adults in the community.

**Methods:**

This is a longitudinal study based on data collection in 2015 and 2020. Descriptive analysis and Structural Equation Modeling were performed, considering p<0.05.

**Results:**

The sample consisted of 220 older adults with a mean baseline age of 68.86 years (± 7.10). Direct associations of higher BMI value with younger age, higher monthly family income, greater functional disability to perform instrumental activities of daily living and greater number of morbidities were found. In addition, education, gender, moderate to vigorous physical activity were indirectly associated with high BMI.

**Conclusion:**

These findings provide insights into the complex relationship of multiple determinants of nutritional status in older adults and support the design of public health policies that consider the specificities of this population group.

## Introduction

Overweight and obesity, determined from a high body mass index (BMI), have been the cause of pressing concern in developing countries due to the disproportionate consumption of low-cost foods with high energy density and the increasingly sedentary lifestyle [1]. Recent evidence demonstrates that a higher BMI value has been associated with type 2 diabetes, cognitive impairment, and poorer quality of life [2, 3]. These data reinforce the need to understand the determinants that influence the BMI of older adults, which would make it possible to monitor the nutritional status of this population.

It is noteworthy that the use of anthropometric measures, such as BMI, has been recommended for the assessment of the nutritional status of older adults in populational studies, as it is a simple method to use, in addition of being easy to interpret and having fewer cultural limitations [4]. Although it is fundamental for the design of public policies, the identification of factors associated with the change in the nutritional status of older adults population is considered complex due to changes in the human aging process, linked to sociodemographic, behavioral and health determinants that can interfere with the nutritional status of older adults [1, 5, 6].

In previous research with Japanese older adults, it was shown that different sociodemographic variables (education and income) had distinct and independent effects on changes in BMI during 19 years of follow-up [7]. The interaction of socioeconomic factors such as gender and race in determining the nutritional status of older adults, as found in another study in the USA [8], reinforces the relevance of considering the multiple determinants of BMI in older adults. Furthermore, behavioral and health factors also suffer interference from socioeconomic aspects [8] and can impact older adults BMI. Corroborating this information, the study involving Spanish older adults showed that physical activity was inversely associated with BMI, that is, the shorter the time spent in these activities, the greater the chance of increasing body weight [9]. In another investigation with older adults Americans, it was found that those who remained exposed to sedentary behavior for a shorter time had a lower BMI when compared to those who remained in this behavior for long periods [10].

However, the aforementioned surveys were carried out in high-income countries, reinforcing existing gaps in the scientific literature on factors related to changes in the nutritional status of older adults population, especially in developing countries [1]. It is noteworthy that, until this date, have not had been identified longitudinal studies, conducted in Brazil, which have verified the explanatory factors of the relationship between sociodemographic, behavioral and health conditions variables on the BMI of older adults, through models previously tested in mediation analysis, that is, indirect relationships.

Considering the dependence and interrelationship of multiple determinants of BMI in older adults, analyses are needed that estimate direct and mediated effects among the variables that make up the causal network of the outcome of interest, such as structural equation modeling (SEM) analysis [11]. Based on the results of previous researches [5, 7, 8], it is assumed that the higher BMI results from sociodemographic, behavioral and health conditions of older adults. Thus, the aim of this study was to verify direct and indirect effects of sociodemographic, behavioral and health conditions variables on the BMI of older adults in the community.

## Material and methods

### Study design and location

The current research is part of a larger project entitled “Estudo Longitudinal de Saúde do Idoso de Alcobaça” (ELSIA). This is a study with a quantitative approach, of the household survey type, longitudinal, developed in the urban area of the municipality of Alcobaça (Bahia)—Brazil, with data collection in 2015 (baseline) and 2020 (follow-up).

To present the findings of the current study, was used the recommendations of the Checklist for Reporting Results of Internet E-Surveys guidelines and Strengthening the Reporting of Observational Studies in Epidemiology (STROBE).

### Study population and sample

The study population consisted of older adults registered in the Family Health Strategy (FHS) in the municipality of Alcobaça, Bahia. For the current research sample, the following inclusion criteria were considered: older adults aged 60 years or over, who lived in the urban area of that city and who were interviewed in both waves (2015 and 2020). Institutionalized older adults were excluded; with communication problems such as deafness, not corrected by devices, severe speech and/or hearing disorders; and/or with cognitive decline.

**Fig 1:**
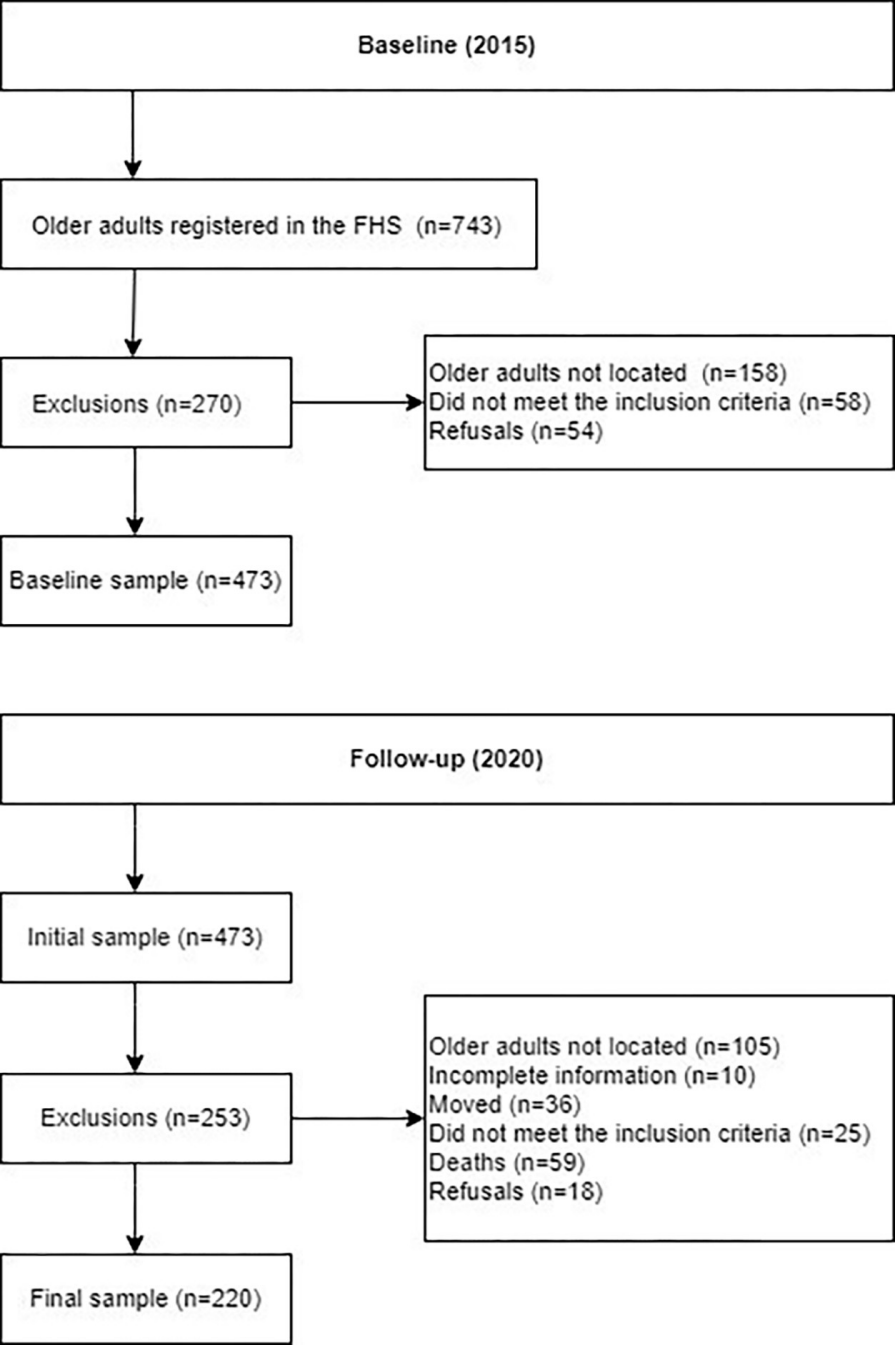
Flowchart for the selection of the sample of older adults residing in the municipality of Alcobaça, Bahia, Brazil, 2015–2020.

In 2015, 743 older adults were registered with the FHS and were invited to participate in the current study, as detailed in previous research [12]. Based on the eligibility criteria, 452 older adults were interviewed during this period. Later, in 2020, older adults located and who met the inclusion criteria were interviewed again. Thus, the final sample of the present investigation consisted of 220 older adults, as shown in Fig 1.

### Data collection procedures and instruments

Data were collected through direct interviews, in older adults´ homes, from July to October 2015 (baseline) and from January to February 2020 (follow-up). At both times, the selected interviewers, with previous experience, received training on how to complete the data collection instruments, and how to approach the participant and ethical issues in conducting the research.

Cognitive decline was assessed by the Mini Mental State Examination, considering a score equal to or greater than 12 points. Sociodemographic data, morbidities and the occurrence of falls in the last 12 months were obtained through the application of a structured questionnaire prepared by the researchers.

To measure the weight (kilograms), a portable digital electronic scale, platform type, brand WISO W721 was used with older adults barefoot and wearing light clothing. Height (meters) was measured using a flexible and inelastic measuring tape, 1.5 meters long, divided into centimeters and subdivided into millimeters, fixed on the wall in a flat and regular place, without a baseboard. It was measured with older adults man barefoot, in an orthostatic position with feet together, with his back to the marker, with his eyes on the horizon. BMI was calculated in kg/m^2^.

The practice of physical activity was assessed using the long version of the International Physical Activity Questionnaire (IPAQ), validated for older adults Brazilians [13, 14]. The IPAQ evaluates the time of physical activity, in a usual week, considering moderate to vigorous physical activities (MVPA) developed at work, at home (household tasks), activities performed as means of transport or even those related to recreation, sport, exercise and leisure, practiced for at least 10 continuous minutes. The total MVPA weekly time was determined from the sum of the time of vigorous physical activity, multiplied by 2, with the time of moderate physical activity as performed in a previous study [12]. Total MVPA time was presented in minutes per week.

Sedentary behavior was determined according to the total sitting time (minutes/day) through the weighted average of sitting time on a weekday and a weekend, in accordance with the IPAQ: [(time sitting on a weekday x 5 + sitting time on a weekend day x 2) / 7]. The longer the sitting time, the greater the sedentary behavior.

Data regarding sleep were obtained using the Pittsburgh Sleep Quality Index (PSQI), validated in Brazil [15], which assesses sleep habits over the past month. Total hours of daily sleep were categorized into ≤7 hours/day and >7 hours/day [16].

Functional capacity was assessed based on basic (ADL) and instrumental (IADL) activities of daily living. ADL were measured using the Katz Index, adapted to the Brazilian reality. This scale consists of six items that measure the performance of older adults in self-care activities [17]. For the IADL, the Lawton and Brody Scale, adapted in Brazil, was used, with a score ranging from 7 (highest level of dependence) to 21 points (complete independence), categorizing older adults as total dependent (7 points), partial (8 to 20 points) and independent (21 points) [18]. Performance in activities on each of the scales was considered, with the higher scores for the ADL and the lower for the IADL indicating greater functional disability.

Common mental disorder (CMD) was measured using the Self-Reporting Questionnaire (SRQ-20) [19], which has 20 questions, four related to physical symptoms and 16 to emotional disorders. Older adults answered yes (1 point) or no (0 points) for each question in the questionnaire, considering situations experienced in the last 30 days. The higher the score, the greater the indicative of psychological morbidities.

### Study variables

Predictive variables: gender (female; male), age (mean years of completed life), literacy (mean years of study) and monthly family income (mean number of minimum wages), color/race (white; non-white); health conditions: morbidities (average number of morbidities), occurrence of falls (average number of falls in the last 12 months), CMD (average total score of the SRQ-20), functional capacity (average of the ADL and IADL scores), sleep (≤7 hours/day and >7 hours/day); behavioral: MVPA (average total IPAQ score) and sedentary behavior (average time spent in sedentary behavior in minutes/day). Outcome variable: BMI (mean of the total BMI value).

### Data analysis

Data were tabulated in double entry using EpiData, version 3.1b. Subsequently, data consistency was checked. Analyses were performed using the Statistical Package for Social Sciences (SPSS®), version 24.0 and Analysis of Moment Structures (AMOS®), version 24. Data were submitted to absolute and relative frequency analysis for categorical variables, mean and standard deviation for quantitative ones.

**Fig 2:**
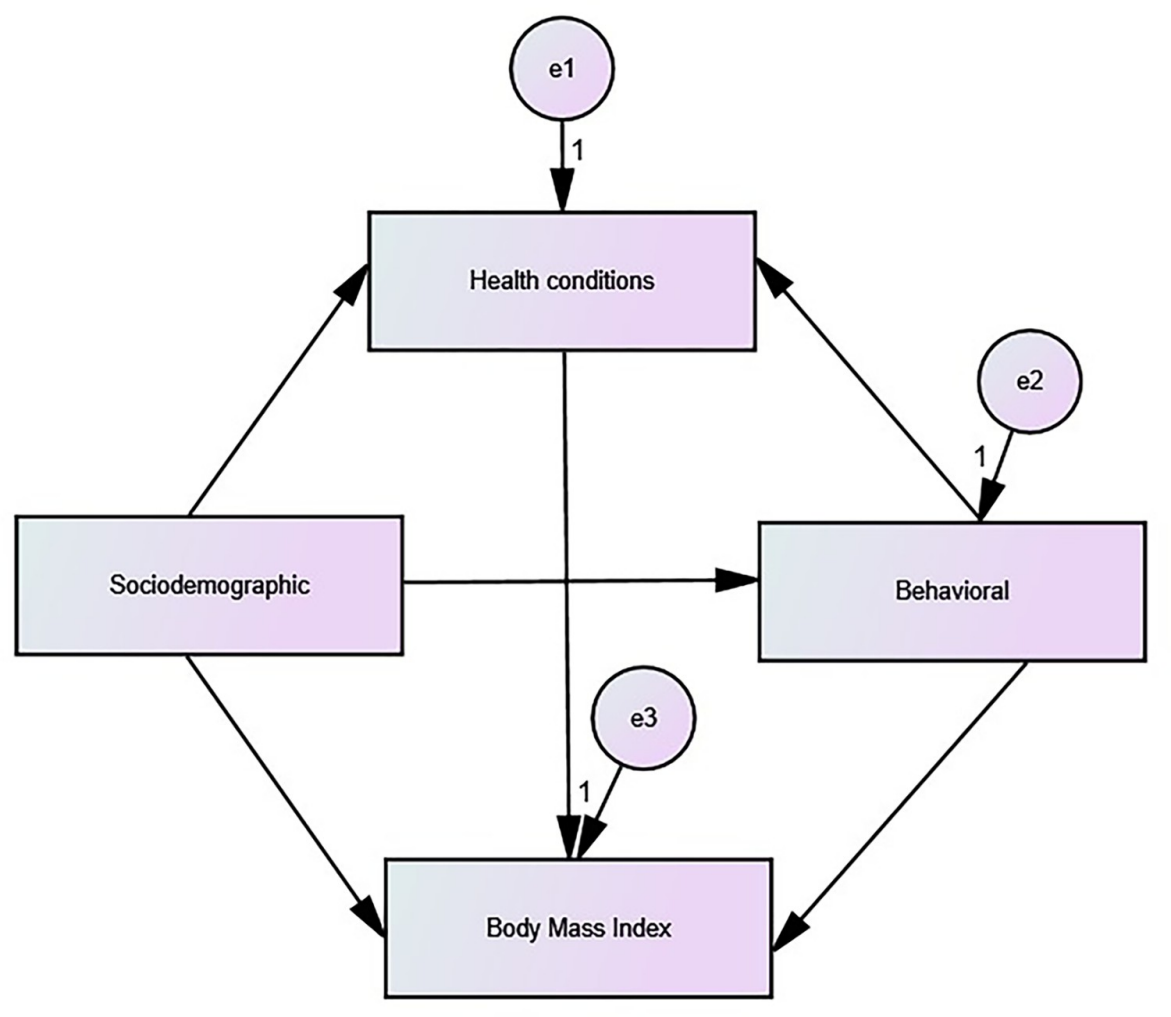
Hypothetical model for analysis of the association between sociodemographic, behavioral and health conditions variables with the body mass index of older adults living in the municipality of Alcobaça, Bahia, Brazil, 2015–2020.

To build the structural model, it was considered that sociodemographic and behavioral characteristics and health conditions are associated with higher BMI through direct and indirect trajectories. Thus, a hypothetical model was elaborated (Fig 2), tested through path analysis [11] and composed of observed variables, represented by rectangles, and classified as endogenous and exogenous. It should be noted that, in the hypothetical model, the endogenous variables receive directional arrows and measurement errors are attributed, specified by “and” [11].

From the specified hypothetical model (Fig 2), the steps for the analysis of SEM were carried out: data collection, model estimation and assessment of the quality of adjustment [11]. The parameters were estimated by the Free Asymptotic Distribution method and the goodness of fit of the models were evaluated according to the Chi-square test (χ^2^) p>0.05; Goodness of Fit Index (GFI) ≥ 0.95; Comparative Fit Index (CFI) ≥ 0.95; Tucker-Lewis Index (TLI) ≥ 0.90 and Root Mean Error of Approximation (RMSEA) ≤ 0.05 (Maroco 2014). Initially, the hypothetical model was tested, and later re-specifications were carried out. For this purpose, non-significant pathways were eliminated (p>0.05) and the change rates (≥11) were calculated [11].

In the analysis of trajectories, the variables: age, monthly family income, education, MVPA, sedentary behavior, morbidities, occurrence of falls, BMI, ADL, IADL and CMD scores were used in a quantitative way. The variables: gender, race/color and sleep were used categorically. For data analysis, baseline predictors were considered; and outcome variable (BMI) of the five-year follow-up.

In the analyzed model, the direct effects were presented through the estimates of the standardized coefficients of the trajectories between sociodemographic and behavioral variables, health conditions and BMI. Furthermore, indirect effects (mediation effects) were determined from the intermediate trajectories between the aforementioned variables. In all tests, type I error was set at 5% (p<0.05).

### Ethical procedures

The baseline survey (2015) was approved by the Research Ethics Committee of the Federal University of Triângulo Mineiro, under number 966.983; while the follow-up by the Research Ethics Committee of the University of the State of Bahia, under number 3,471,114. This study meets the ethical principles of the Declaration of Helsinki and Resolution 466/12 of the National Health Council. All participants agreed to participate in the study and signed the informed consent form.

## Results

Of the total participants in the baseline (n = 220), 64.5% (n = 142) were female, 69.1% (n = 152) declared themselves as non-white, and 55.5% (n = 122) slept for ≤7 hours/day. Table 1 shows other characteristics of older adults participants in the study.

**Table 1:** Characterization of the sample (baseline) of older adults residing in the municipality of Alcobaça, Bahia, Brazil, 2015 (n = 220).

	Mean	SD
Age	68.86	7.10
Literacy	4.21	5.02
Income	2.86	4.86
Morbidities	3.20	2.77
Falls	0.56	1.33
IADL	0.21	0.54
ADL	11.79	2.30
CMD	3.78	3.93
MVPA (minutes/week)	413.67	527.51
SB (minutes/day)	424.33	153.32
BMI	27.90	5.48

SD: Standard deviation; ADL: Basic activities of daily living; IADL: Instrumental activities of daily living; CMD: Common mental disorder; MVPA: Moderate to vigorous physical activity; SB: Sedentary behavior; BMI: Body mass index.

**Fig 3:**
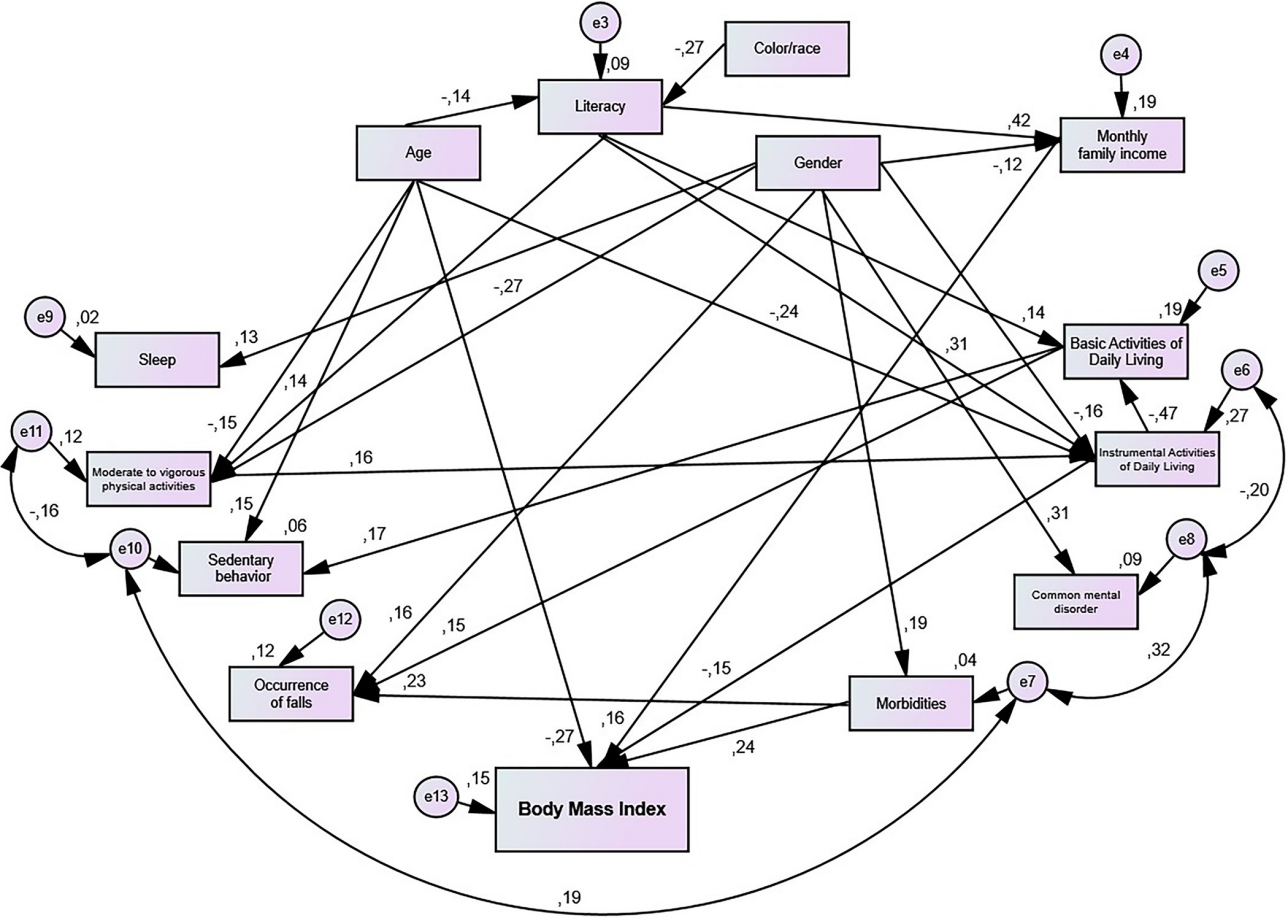
Model for analysis of the direct and indirect effects of sociodemographic, behavioral and health conditions variables on the body mass index of older adults living in the municipality of Alcobaça, Bahia, Brazil, 2015–2020. (x^2^ (df = 62) = 71.0; p = 0.204; CFI = 0.97; GFI = 0.96; TLI = 0.96; RMSEA = 0.03).

Fig 3 shows the direct and indirect effects of sociodemographic, behavioral and health conditions variables on the BMI of older adults people in the community, after a five-year follow-up.

The direct estimators of the associations between sociodemographic, behavioral and health conditions variables with the BMI of older adults living in the municipality of Alcobaça (BA) are shown in Table 2.

**Table 2:** Direct standardized coefficients for variables related to the body mass index of older adults living in the municipality of Alcobaça, Bahia, Brazil, 2015–2020.

Direct effects	Estimator	*p* *
Age	-0.27	<0.001
Income	0.16	0.010
Mobilities	0.24	<0.001
IADL	-0.15	0.022

IADL: Instrumental activities of daily living

*p<0.05

Direct associations were found between younger age (p<0.001), higher monthly family income (p = 0.001), greater functional disability for IADL (p = 0.02) and higher number of morbidities (p<0.001) with the higher BMI value (Table 2).

It was also observed that higher education (β = 0.07) and male gender (β = -0.02), mediated by higher monthly family income, were indirectly associated with the higher total BMI value, such as the shorter MVPA time (β = -0.02) and lower educational level (β = -0.05), mediated by greater functional incapacity for IADL (Fig 2). Furthermore, the female gender, mediated by a greater number of morbidities (β = 0.05), greater functional incapacity for IADL (β = -0.02) and lower monthly family income (β = -0.02), was associated with indirectly to the higher total BMI value among older adults (Fig 2).

## Discussion

Through the analysis of SEM, the current study found direct associations of higher BMI value with younger age, higher monthly family income, greater functional incapacity for IADL and higher number of morbidities. Additionally, it was identified that the variables education, gender, MVPA were indirectly associated with higher BMI.

These results should be carefully analyzed due to limitations regarding the power of generalization (external validity) of the findings for older adults population, considering that older adults people registered in the FHS in the municipality of Alcobaça-BA participated in the study. Nevertheless, the way in which some variables are measured (time spent on physical activity, sedentary behavior and morbidities) through self-report may underestimate or overestimate some of the evidenced information.

On the other hand, the use of SEM analysis allows us to understand the direct and indirect relationship of multiple factors (sociodemographic, behavioral, and health conditions) on BMI in older adults. It is also noteworthy that, until this date, no other longitudinal study conducted in Brazil with this proposal has been identified, reiterating the relevance of this research in advancing knowledge about factors related to changes in the nutritional status of older adults population.

The negative association of younger age with the higher BMI value found in the current study is consistent with other evidence [5, 7, 20], which found an inverse relationship between age and BMI, that is, the younger the age, the greater the value of this anthropometric measure. Such results may be related to changes in body composition that occur with the human aging process, especially after 70 years old [6] Advancing age is accompanied by hormonal changes that reduce protein synthesis, leading to a progressive loss of skeletal muscle mass and strength (sarcopenia) and compromising the functionality of older adults [21]. It is also worth highlighting the reduction in fat mass and its redistribution to the visceral component, skeletal muscle and liver [6], in addition to the reduction in height. These changes, arising from the aging process, can affect the nutritional status of older adults [22] causing younger older adults to have a higher BMI value with a decrease in this quantity over the years, although it should be noted that this anthropometric measure does not allow predicting the distribution of body fat over age, nor does it discriminate the loss of lean mass.

In the current investigation, monthly family income was also directly associated with BMI. A similar result was found in a longitudinal study carried out with older adults Japanese, in which a higher monthly income at baseline was associated with a higher BMI at follow-up [7]. In Brazil, findings from a longitudinal study, using secondary data from the Brazilian Institute of Geography and Statistics, corroborate the current study by showing that older adults with higher incomes had the higher BMI strata (≥25 kg/m2) [4].

One aspect that explains the relationship between income and BMI refers to access to healthy food. A healthy diet in older adults refers to the need to include more expensive foods, such as whole grains, vegetables, fruits and dairy products. Older adults with limited income may have financial difficulties, which would impact the purchase of foods that ensure the quality of the diet and, consequently, lead to weight loss during the aging process [23].

It should be noted that the protective effects of income on the nutritional status of older adults are not considered universal and are related to other contextual factors [1]. In developing countries, such as Brazil, there has been a tendency in recent years to change the nutritional status of older adults population due to the disproportionate consumption of foods with high energy density, linked to a more sedentary lifestyle [8].

As an effect of the nutritional transition, individuals with better socioeconomic status changed their eating pattern by replacing healthier foods with high-calorie, low-nutrient foods, as this group has greater access and availability to industrialized products [24]. However, this phenomenon has still been little explored in the national gerontological literature, which denotes the need to deep the knowledge about the influence of income on the nutritional status of older adults Brazilians [25].

In addition to socioeconomic factors, the greater functional incapacity for IADL was directly associated with the higher BMI value among older adults in the current investigation. Data from a population study that analyzed older adults Swedish people support this finding when they found that those with difficulty in performing activities of daily living were more likely to have a BMI ≥30 kg/m^2^ when compared to those who did not have this condition [26]. These results were also observed in a survey conducted in Brazil in which most older adults with functional dependence had a BMI >27 kg/m^2^ [27]. Older adults with functional dependence tend to perform low-energy activities in the home context [27], which can contribute to increased body weight.

In the current investigation, it was also found that the higher number of morbidities was directly associated with the higher BMI value; in line with another longitudinal survey conducted with older adults Japanese, whose higher burden of disease was associated with a higher level of BMI (β = 0.149, p = 0.001) [7]. In China, it was shown that middle-aged and older adults who had any type of chronic disease were more likely to have a higher BMI [28].

The acceptance of the health problem by older adults is essential for self-care as it influences the search for health services and, therefore, their treatment [29]. However, in the prospective cohort study carried out in southern Brazil, it was identified that after three years of follow-up, older adults people with chronic diseases did not change their food intake in order to collaborate in the secondary treatment of these conditions [30]. Therefore, it is inferred that even after the diagnosis of diseases, older adults tend to maintain an inappropriate lifestyle, which could cause health problems and possibly interfere with the evolution of the BMI over the years.

The indirect association of higher educational level, mediated by higher monthly family income, with a higher BMI value promotes the result of a research carried out in Japan, in which the authors found that the higher educational level of older adults was correlated with higher values of family income at baseline and also acted as a predictor for higher BMI during follow-up [7].

Levels of education and income are the main indicators of the individual’s socioeconomic position and reflect on healthy behaviors, as well as on health conditions and psychosocial well-being. Both variables, therefore, are considered determinants for the change in BMI in older adults over the course of the human aging process [8]. However, the findings of the current study reinforce the hypothesis that income is the reason why education impacts the nutritional status of older adults [8].

This result can be explained by the fact that older adults with higher education tend to have higher family income, which could possibly influence adherence to healthy behaviors during the aging process, such as regular physical activity and healthier eating habits [31, 32]. Furthermore, it is highlighted that the variables, income and education, can serve as a proxy for a variety of different constructs not measured in this study and that influences the nutritional status of older adults, including contextual factors (social vulnerability), access to health services and healthy eating.

In the current study, the higher monthly family income also mediated the association of males with the higher BMI value. Despite having assessed the nutritional status of older adults using another indicator, a survey carried out in Indonesia partially supports the findings of the present study by identifying a positive correlation between the concentration of distribution of older adults males with waist circumference greater than 90 cm and the income indicator (poor socioeconomic status; physical work; and lack of formal education) (p<0.001) [24].

It is noteworthy that in the current investigation, the relationship between income and BMI differed with regard to gender. Among older adults, it was found that the lower monthly family income mediated the association of the higher BMI value, unlike males. Similar data were evidenced in another longitudinal survey conducted with older adults in Canada, in which higher income reduced the risk of women presenting higher strata of nutritional status classification (BMI ≥25 kg/m^2^) [33]. However, in an investigation carried out in Brazil, a divergent result was found when observing an increase in the prevalence of overweight (25 ≤ BMI < 30 kg/m^2^) and obesity (BMI ≥30 kg/m^2^) among older adult women with higher income levels [4].

Physiologically, women tend to increase their body weight due to changes in the metabolic profile that are common in the aging process, modifying the composition and distribution of body adipose tissue [34]. However, the findings of the current study foster the understanding that sociodemographic determinants also influence the nutritional status of older adults and differ according to gender [33]. Although women, regardless of socioeconomic status, are more likely to have greater changes in body composition when compared to men, some studies highlight that the higher prevalence of obesity in females has affected lower-income groups [24].

Also in relation to females, it was also identified that health variables (higher number of morbidities and functional incapacity for IADL) mediated the association with the higher BMI value. In a longitudinal study carried out in China, it was observed, among older women, that the absence of severe disease and functional independence constituted a protective factor for BMI ≥28.0 kg/m^2^ [35], supporting in parts the data from the present study.

As a result of their longer life expectancy, older women are more vulnerable to functional dependence [36] and chronic diseases [37] compared to men; and these health outcomes can impact their nutritional status [7, 27] during the aging process.

The shorter MVPA time associated with the higher BMI value supports the evidence in the literature [9, 26]. In the cross-sectional study carried out with Swedish older people, it was found that the longer MVPA, also measured by the IPAQ, was a protective factor for the occurrence of BMI ≥30.0 kg/m^2^ [26]. In the prospective cohort investigation carried out with older adults in Spain, it was possible to identify that physical activity, in the leisure context, was an independent variable and inversely associated with BMI, both in the total assessment and in the analysis stratified by intensity levels [9].

It is noteworthy that, in the current study, the association between these variables (MVPA and BMI) was indirect and mediated by greater functional incapacity for IADL. This result partially corroborates with a research carried out in Sweden in which MVPA was statistically associated with BMI ≥30.0 kg/m^2^ among older adults with functional decline, even after adjusting for other variables (gender, age, socioeconomic level and consumption of fruits and vegetables) [26].

Physical activity contributes to attenuating the harmful effects of inflammation on the physical mobility of older adults with changes in nutritional status [38]. In addition to the anti-inflammatory effect, regular physical activity helps with mobility, flexibility and maintenance of muscle strength [38]. All these factors contribute to the prevention of functional decline with advancing age and, consequently, impact on metabolic processes, which involve the body composition of older adults [38, 39].

Linked to this, lower educational level, mediated by greater functional incapacity for IADL, was also indirectly associated with higher BMI. IADL are considered activities that are more complex and require cognitive skills that are influenced by low education [40]. Older adults with functional dependence spend most of their time sitting or lying down and occasionally take light walks in their own home context [27], which could alter their nutritional status.

One of the greater challenges for the treatment of changes in the nutritional status of older adults is the preservation of muscle mass [41]. Thus, lifestyle interventions, which include healthy eating and regular physical activity, should prioritize the maintenance of muscle mass, in addition to the loss of body weight [6].

From this study, it was observed that younger age, higher monthly income, greater functional incapacity for IADL and a greater number of morbidities were directly associated with a higher BMI value. It was found that higher education and male gender, mediated by higher monthly income, were indirectly associated with higher total BMI, as well as shorter MVPA and lower education, mediated by higher functional incapacity for IADL. Furthermore, the female gender, mediated by a greater number of morbidities, greater functional incapacity for IADL and lower monthly income, was indirectly associated with the higher total BMI value among older adults.

The findings of this study make it possible to understand BMI in older adults with structural equation modeling, providing insights into the complex relationship of multiple determinants of nutritional status in older adults population. It is inferred that this evidence can support the design of public health policies in favor of monitoring the nutritional status of older adults population, considering the specificities of this population segment.

## References

[1] Malenfant JH, Batsis JA. Obesity in the geriatric population–a global health perspective. J Glob Heal reports. 2019;3. doi: 10.29392/joghr.3.e2019045 34027129 PMC8136402

[2] Saltiel AR, Olefsky JM. Inflammatory mechanisms linking obesity and metabolic disease. J Clin Invest. 2017;127(1):1–4. doi: 10.1172/JCI92035 28045402 PMC5199709

[3] Rambod M, Ghodsbin F, Moradi A. The Association Between Body Mass Index and Comorbidity, Quality of Life, and Cognitive Function in the Elderly Population. Int J Community Based Nurs Midwifery. 2020 Dec 1;8(1):45. doi: 10.30476/IJCBNM.2019.81677.0 32039279 PMC6969950

[4] da Silva VS, Souza I, Silva DAS, Barbosa AR, da Fonseca M de JM. Evolução e associação do IMC entre variáveis sociodemográficas e de condições de vida em idosos do Brasil: 2002/03-2008/09. Cien Saude Colet. 2018 Mar 1;23(3):891–901. 29538569 10.1590/1413-81232018233.12532016

[5] Pereira IF da S, Spyrides MHC, Andrade L de MB. Estado nutricional de idosos no Brasil: uma abordagem multinível. Cad Saude Publica. 2016 Jun 3;32(5):e00178814. 10.1590/0102-311X00178814 27276697

[6] Mckee A, and Morley J. Obesity in the Elderly. In: Endotext, editor. MDText.com. South Dartmouth (MA): MDText.com, Inc.; 2000; 2021.

[7] Murayama H, Liang J, Bennett JM, Shaw BA, Botoseneanu A, Kobayashi E, et al. Socioeconomic Status and the Trajectory of Body Mass Index Among Older Japanese: A Nationwide Cohort Study of 1987–2006. J Gerontol B Psychol Sci Soc Sci. 2016 Mar 1;71(2):378–88. doi: 10.1093/geronb/gbu183 25577567 PMC5926508

[8] Assari S, Cobb S BM. Race by Gender Differences in the Protective Effects of Education and Income Against Subsequent Changes in Self-rated Health, Physical Activity, and Body Mass Index Among Older Americans. J Heal Econ Dev. 2019;1(2):9–21. 32201864

[9] Cárdenas Fuentes G, Bawaked RA, González MÁM, Corella D, Cachinero IS, Salas-Salvadó J, et al. Association of physical activity with body mass index, waist circumference and incidence of obesity in older adults. Eur J Public Health. 2018 Oct 1;28(5):944–50. doi: 10.1093/eurpub/cky030 29554269

[10] Bann D, Hire D, Manini T, Cooper R, Botoseneanu A, McDermott MM, et al. Light Intensity physical activity and sedentary behavior in relation to body mass index and grip strength in older adults: cross-sectional findings from the Lifestyle Interventions and Independence for Elders (LIFE) study. PLoS One. 2015 Feb;10(2). 10.1371/journal.pone.0116058 PMC4315494 25647685

[11] Marôco J. Análise de Equações Estruturais: Fundamentos teóricos, software & Aplicações. ReportNumber L, editor. Perô Pinheiro: Report Number, Lda; 2014.

[12] Camilo B de F, Meneguci J, Tribess S, Virtuoso Júnior JS, Damião R. Associação combinada e independente do comportamento sedentário e atividade física com sobrepeso e obesidade em idosos. Rev Iberoam Psicol del Ejerc y el Deport. 2019;1(15):19–25.

[13] Benedetti TRB, Antunes PDC, Rodriguez-Añez CR, Mazo GZ, Petroski ÉL. Reprodutibilidade e validade do Questionário Internacional de Atividade Física (IPAQ) em homens idosos. Rev Bras Med do Esporte. 2007;13(1):11–6.

[14] Benedetti T, Mazo GZ, Barros MVG. Aplicação do Questionário Internacional de Atividades Físicas para avaliação do nível de atividades físicas de mulheres idosas: validade concorrente e reprodutibilidade teste-reteste. Rev Bras Ciência e Mov. 2004;12(1):25–34.

[15] Bertolazi AN, Fagondes SC, Hoff LS, Dartora EG, da Silva Miozzo IC, de Barba MEF, et al. Validation of the Brazilian Portuguese version of the Pittsburgh Sleep Quality Index. Sleep Med. 2011;12(1):70–5. doi: 10.1016/j.sleep.2010.04.020 21145786

[16] Wei J, Hou R, Xie L, Chandrasekar EK, Lu H, Wang T, et al. Sleep, sedentary activity, physical activity, and cognitive function among older adults: The National Health and Nutrition Examination Survey, 2011–2014. J Sci Med Sport. 2021 Feb 1;24(2):189–94. doi: 10.1016/j.jsams.2020.09.013 33032913

[17] Lino VTS, Pereira SRM, Camacho LAB, Ribeiro Filho ST, Buksman S. Adaptação transcultural da Escala de Independência em Atividades da Vida Diária (Escala de Katz). Cad Saude Publica. 2008;24(1):103–12. 18209838 10.1590/s0102-311x2008000100010

[18] RL S, Virtuoso Júnior JS. Reliability of the Brazilian version of the Scale of Instrumental Activities of Daily Living. Rbps. 2008;21(4):290–6.

[19] Scazufca M, Menezes PR, Vallada H, Araya R. Validity of the self reporting questionnaire-20 in epidemiological studies with older adults: results from the Sao Paulo Ageing & Health Study. Soc Psychiatry Psychiatr Epidemiol. 2009;44(3):247–54. 18777144 10.1007/s00127-008-0425-y

[20] Kong JW, Park T, Lee DR, Lee J. Trajectories of Body Mass Index and Their Associations with Mortality among Older Adults in Korea: Analysis of the Korean Longitudinal Study of Aging. Ann Geriatr Med Res. 2020;24(3):195. doi: 10.4235/agmr.20.0030 32829573 PMC7533191

[21] Walston JD. Sarcopenia in older adults. Curr Opin Rheumatol. 2012 Nov;24(6):623. doi: 10.1097/BOR.0b013e328358d59b 22955023 PMC4066461

[22] Batsis JA, Villareal DT. Sarcopenic obesity in older adults: aetiology, epidemiology and treatment strategies. Nat Rev Endocrinol. 2018 Sep 1;14(9):513–37. doi: 10.1038/s41574-018-0062-9 30065268 PMC6241236

[23] Gomes AP, Soares ALG, Gonçalves H. Baixa qualidade da dieta de idosos: estudo de base populacional no sul do Brasil. Cien Saude Colet. 2016 Nov 1;21(11):3417–28. doi: 10.1590/1413-812320152111.17502015 27828575

[24] Pujilestari CU, Nyström L, Norberg M, Weinehall L, Hakimi M, Ng N. Socioeconomic inequality in abdominal obesity among older people in Purworejo District, Central Java, Indonesia-A decomposition analysis approach. Int J Equity Health. 2017 Dec 12;16(1):1–11. 29233136 10.1186/s12939-017-0708-6 PMC5727959

[25] Tavares DM dos S, Bolina AF, Dias FA, Ferreira PC dos S, Santos NM de F. Excesso de peso em idosos rurais: associação com as condições de saúde e qualidade de vida. Cien Saude Colet. 2018 Mar 1;23(3):913–22. 29538571 10.1590/1413-81232018233.25492015

[26] Asp M, Simonsson B, Larm P, Molarius A. Physical mobility, physical activity, and obesity among elderly: findings from a large population-based Swedish survey. Public Health. 2017 Jun 1;147:84–91. 28404503 10.1016/j.puhe.2017.01.032

[27] Lima PV, Lopes AOS, Duarte SFP, Oliveira AS de, Correa GTB, Reis LA Dos. Profile of the body mass index and associated factors in active elderlies. Rev Bras Enferm. 2018;71:876–83. doi: 10.1590/0034-7167-2016-0683 29791622

[28] Wang R, Feng Z, Xue D, Liu Y, Wu R. Exploring the links between population density, lifestyle, and being overweight: Secondary data analyses of middle-aged and older Chinese adults. Health Qual Life Outcomes. 2019 Jun 11;17(1):1–10. 31186044 10.1186/s12955-019-1172-3 PMC6558806

[29] Bolina AF, Pegorari MS, Tavares DM dos S. Obesity awareness among elders living in rural area: a household survey. Rev Bras Cineantropometria Desempenho Hum. 2017;19(5):565–74.

[30] Cembranel F, Bernardo C de O, Ozcariz SGI, D’Orsi E. Impact of the diagnosis of diabetes and/or hypertension on healthy food consumption indicators: a longitudinal study of elderly persons. Rev Bras Geriatr e Gerontol. 2017 Feb;20(1):33–44.

[31] Dumith SC, Maciel FV, Borchardt JL, Alam VS, Silveira FC, Paulitsch RG. Preditores e condições de saúde associados à prática de atividade física moderada e vigorosa em adultos e idosos no sul do Brasil. Rev Bras Epidemiol. 2019 Mar 21;22:e190023. 30916145 10.1590/1980-549720190023

[32] Gomes AP, Bierhals IO, Vieira LS, Soares ALG, Flores TR, Assunção MCF, et al. Padrões alimentares de idosos e seus determinantes: estudo de base populacional no sul do Brasil. Cien Saude Colet. 2020 Jun 3;25(6):1999–2008. 32520248 10.1590/1413-81232020256.20932018

[33] Wang M, Yi Y, Roebothan B, Colbourne J, Maddalena V, Wang PP, et al. Body Mass Index Trajectories among Middle-Aged and Elderly Canadians and Associated Health Outcomes. J Environ Public Health. 2016;2016. doi: 10.1155/2016/7014857 26925112 PMC4748085

[34] Melo JB de, Campos RCA, Carvalho PC, Meireles MF, Andrade MVG, Rocha TPO, et al. Cardiovascular Risk Factors in Climacteric Women with Coronary Artery Disease. Int J Cardiovasc Sci. 2017;31(1):4–11.

[35] Luo H, Ren X, Li J, Wu K, Wang Y, Chen Q, et al. Association between obesity status and successful aging among older people in China: Evidence from CHARLS. BMC Public Health. 2020 May 24;20(1):1–10. 32448262 10.1186/s12889-020-08899-9 PMC7245862

[36] Saito J, Kondo N, Saito M, Takagi D, Tani Y, Haseda M, et al. Exploring 2.5-Year Trajectories of Functional Decline in Older Adults by Applying a Growth Mixture Model and Frequency of Outings as a Predictor: A 2010–2013 JAGES Longitudinal Study. J Epidemiol. 2019 Feb 5;29(2):65–72. doi: 10.2188/jea.JE20170230 29937470 PMC6336721

[37] Mini GK, Thankappan KR. Pattern, correlates and implications of non-communicable disease multimorbidity among older adults in selected Indian states: a cross-sectional study. BMJ Open. 2017 Mar 1;7(3). doi: 10.1136/bmjopen-2016-013529 28274966 PMC5353268

[38] Tay J, Goss AM, Locher JL, Ard JD, Gower BA. Physical Function and Strength in Relation to Inflammation in Older Adults with Obesity and Increased Cardiometabolic Risk. J Nutr Health Aging. 2019 Dec 1;23(10):949–57. doi: 10.1007/s12603-019-1260-4 31781724 PMC6996491

[39] Rosique-Esteban N, Babio N, Díaz-López A, Romaguera D, Alfredo Martínez J, Sanchez VM, et al. Leisure-time physical activity at moderate and high intensity is associated with parameters of body composition, muscle strength and sarcopenia in aged adults with obesity and metabolic syndrome from the PREDIMED-Plus study. Clin Nutr. 2019 Jun 1;38(3):1324–31. doi: 10.1016/j.clnu.2018.05.023 29910068

[40] Brigola AG, Alexandre TDS, Inouye K, Yassuda MS, Pavarini SCI, Mioshi E. Limited formal education is strongly associated with lower cognitive status, functional disability and frailty status in older adults. Dement Neuropsychol. 2019 Apr 1; 13(2):216. doi: 10.1590/1980-57642018dn13-020011 31285797 PMC6601310

[41] Camilo BF. Atividade física, comportamento sedentário e excesso de peso em idosos: um estudo longitudinal. Tese de Doutorado, Universidade Federal do Triângulo Mineiro. 2021. Available from: https://bdtd.uftm.edu.br/bitstream/123456789/1161/1/Tese%20Bruno%20F%20Camilo.pdf

